# The Microscopist, or a Complete Manual on the Use of the Microscope

**Published:** 1852-01

**Authors:** 


### ARTICLE VIII.

*The Microscopist or a Complete Manual on the use of the Microscope for Physicians, Students, and all lovers of Natural Science.* With illustrations by JOSEPH H. WYTHES, M. D. Pages 191 duodecimo. Philadelphia: Lindsay & Blakiston, 1851. (From the Publishers through S. C. Griggs & Company.)

As the revelations made by the aid that the microscope has given, by which much of the imperfection of the sense of sight is removed, have opened a new field of study and investigation to the physician as well as the naturalist, the little work before us may be considered quite opportune in its advent. It gives plain and practical lessons in the use of the instrument by which the student may qualify himself to carry on microscopic examinations with facility and satisfaction.

The brief accounts of the cell doctrine of physiology, of the examination of morbid structures and of urinary deposites will be found quite interesting to those physicians who have not seen and studied more elaborate works, upon these subjects. In fact even to those who have not the instrument it may be a very interesting work as it enables them to understand the structure of the microscope and many of the uses that it subserves to the cause of science.

